# An improved preparation pulse for quantitative t2 mapping of blood in the cardiac chambers

**DOI:** 10.1186/1532-429X-17-S1-W31

**Published:** 2015-02-03

**Authors:** Juliet Varghese, Ning Jin, Georgeta Mihai, Orlando P Simonetti

**Affiliations:** 1 Dorothy M. Davis Heart and Lung Research Institute, The Ohio State University Wexner Medical Center, Columbus, OH, USA; 2 Department of Biomedical Engineering, The Ohio State University, Columbus, OH, USA; 3 Siemens Healthcare, Columbus, OH, USA; 4 Department of Radiology, The Ohio State University Wexner Medical Center, Columbus, OH, USA; 5 Division of Cardiovascular Medicine, Department of Internal Medicine, The Ohio State University Wexner Medical Center, Columbus, OH, USA

## Background

T2 is sensitive to hemoglobin oxygen saturation (%HbO2). Non-invasive, rapid in-vivo quantification of %HbO2 based on the T2 of blood may be useful in patients with congenital heart disease. Although single-shot, T2-prepared SSFP enables rapid myocardial T2 quantification [1], flow sensitivity of the T2 preparation, especially at later echo times, may cause an underestimation of T2 values in flowing blood. We aim to reduce flow sensitivity of the T2 preparation pulse for rapid and accurate quantification of T2 in blood.

## Methods

Malcolm-Levitt (MLEV) T2 preparation pulses were implemented with two different schemes: 4 (MLEV4) and 8 refocusing pulses (MLEV8). Quantitative T2 maps (TR = 3099-3712 ms, FA = 40^0^, 2.5x2x8 mm^3^, 1NEX, free breathing), were acquired on a 1.5T MRI system (Avanto, Siemens Healthcare) using both schemes with preparation times: TE_T2p_ = 0,30/33,40,60,80,100,120,140,160,180 and 200 ms. Multi-Echo Spin Echo (MESE) (TR = 10 sec, 16 TEs - 14.4 to 230.4 ms) was used to measure T2 in a phantom with blood-mimicking fluid as a reference standard. Quantitative T2 values were measured in the same phantom and in the right (RV) and left (LV) ventricles in a four-chamber view in five volunteers (mean age: 28 ± 14 years). Paired t-tests were performed (p < 0.05 significant) to assess significant differences between RV and LV T2, and between the different preparation pulses.

## Results

**Figure 1:**
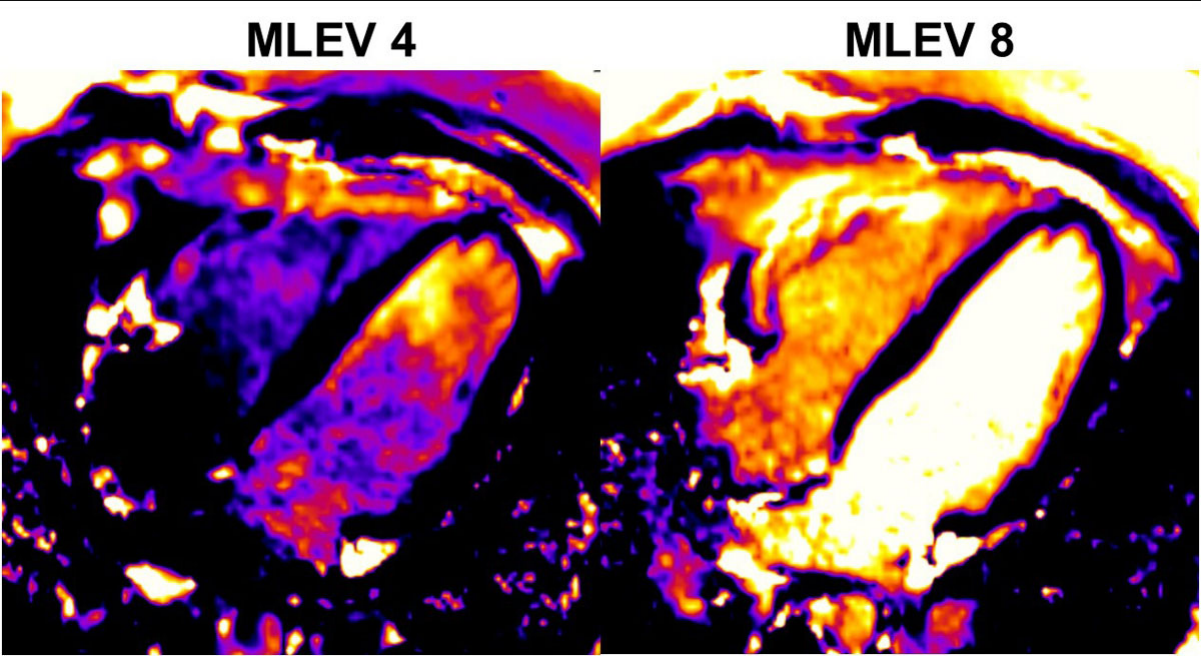
Quantitative T2 maps acquired in a volunteer (4-chamber view) with MLEV4 (left) and MLEV8 (right) T2 preparation schemes.

Phantom T2 values were: MESE T2 = 130 ms, MLEV4 = 101 ms and MLEV8 = 135 ms. Images from a volunteer are shown in Figure 1. The mean ± SD, and coefficient of variation (CV) of T2 values of all volunteers are shown in Table 1. The T2 values for arterial (LV) and venous (RV) blood measured using the MLEV8 preparation were higher than those obtained using MLEV4 and were comparable to previously reported values [2]. A larger difference (p = 0.005) between arterial and venous T2 were observed with MLEV8, along with increased homogeneity as indicated by the lower maximum standard deviation of T2 within the regions of interest (ROI). CV, while similar for the two preparations in the RV, was significantly reduced in the LV using the MLEV8 pulse.

**Table 1:** Mean ± SD and range of T2 values measured in all volunteers in RV and LV with MLEV4 and MLEV8 T2 preparation schemes. Reduced maximum SD shows lower variability within the ROI for MLEV8.

	MLEV4		MLEV8	
	
	RV	LV	RV	LV
Mean ± SD (ms) across subjects	152.36 ± 14.55	160.72 ± 31.17	185.26 ± 18.64*	224.44 ± 12.41*Ï¯

Coefficient of Variation across subjects	9.55	19.4	10.06	5.53

Maximum SD within each ROI across subjects	21.3	21.9	15.7	17.9

*RV and LV T2 with MLEV8 are significantly higher compared to RV and LV T2 with MLEV4.

Ï® LV T2 is significantly different from RV T2 with MLEV8.

## Conclusions

The standard T2 preparation pulse uses 4 refocusing pulses; wide pulse spacing increases flow sensitivity and leads to heterogeneity and signal loss in the blood pool at longer preparation times. We doubled the number of refocusing pulses to keep the pulse spacing short and minimize signal loss due to flow. Our preliminary results show increased T2 homogeneity within the LV blood pool, greater difference between venous and arterial T2, and blood T2 values that more closely match those previously reported. In future studies, the MLEV8 pulse will be further explored in vitro and in vivo for accurate calibration and estimation of %HbO2 from quantitative T2 maps.

## Funding

AHA 13PRE16950001.

## References

[B1] Giri S J Cardiovasc Magn Reson 2009 11 56 10.1186/1532-429X-11-56 20042111 PMC2809052

[B2] Wright GA J Magn Reson Imaging 1991 1 275 283 10.1002/jmri.1880010303 1802140

